# PLGA nanocapsules as a delivery system for a recombinant LRP‐based therapeutic

**DOI:** 10.1002/2211-5463.13809

**Published:** 2024-05-03

**Authors:** Martin Bernert, Monique J. Bignoux, Chandni Madhav, Sichumiso Gqeba, Tyrone C. Otgaar, Gavin Morris, Stefan F. T. Weiss, Eloise Ferreira

**Affiliations:** ^1^ School of Molecular and Cell Biology University of the Witwatersrand Johannesburg South Africa

**Keywords:** LRP, nanoparticle, PLGA, telomerase, therapeutic

## Abstract

Telomerase activity is directly affected by the laminin receptor precursor (LRP) protein, a highly conserved nonintegrin transmembrane receptor, which has been shown to have therapeutic effects in ageing, and age‐related diseases. Recently, it has been found that overexpression of LRP‐FLAG, by plasmid transfection, leads to a significant increase in telomerase activity in cell culture models. This may indicate that upregulation of LRP can be used to treat various age‐related diseases. However, transfection is not a viable treatment strategy for patients. Therefore, we present a nanoencapsulated protein‐based drug synthesised using poly(lactic‐co‐glycolic acid) (PLGA) nanocapsules for delivery of the 37 kDa LRP protein therapeutic. PLGA nanocapsules were synthesised using the double emulsification‐solvent evaporation technique. Different purification methods, including filtration and centrifugation, were tested to ensure that the nanocapsules were within the optimal size range, and the BCA assay was used to determine encapsulation efficiency. The completed drug was tested in a HEK‐293 cell culture model, to investigate the effect on cell viability, LRP protein levels and telomerase activity. A significant increase in total LRP protein levels with a concomitant increase in cell viability and telomerase activity was observed. Due to the observed increase in telomerase activity, this approach could represent a safer alternative to plasmid transfection for the treatment of age‐related diseases.

## Graphical abstract

The laminin receptor precursor (LRP) directly influences telomerase activity, exerting therapeutic benefits in ageing and age‐related diseases. A poly(lactic‐co‐glycolic acid) nanocapsule‐based delivery system was engineered for delivery of the LRP protein, and increased telomerase activity and enhanced cell viability was observed in mammalian cells, confirming its therapeutic efficacy. This LRP‐based therapeutic could therefore potentially aid in the treatment of age‐related diseases.

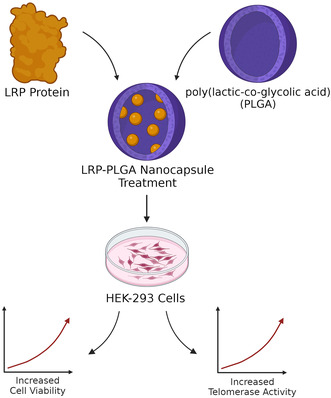

**Abbreviations**
BCA: bicinchoninic acid
BSA: bovine serum albumin
CHAPS: 3‐((3‐cholamidopropyl) dimethylammonio)‐1‐propanesulfonate
DCM: dichloromethane
DLS: dynamic light scattering
DMEM: Dulbecco's Modified Eagle's Medium
DMSO: dimethyl sulfoxide
DNA: deoxyribonucleic acid
EA: ethyl acetate
EGTA: ethylene glycol‐bis (β‐aminoethyl ether)‐*N*,*N*,*N*′,*N*′‐tetraacetic acid
FBS: fetal bovine serum
FDA: USA food and drug administration
HEK‐293: human embryonic kidney
hTERC: human telomerase RNA component
hTERT: human telomerase reverse transcriptase
LRP: laminin receptor precursor
LRP/LR: 37 kDa laminin receptor precursor/67 kDa high affinity laminin receptor
MTT: 3‐(4,5‐dimethylthiazol‐2‐yl)‐2,5‐diphenyltetrazolium bromide
PBS: phosphate‐buffered saline
PBST: phosphate‐buffered saline with tween
PCR: polymerase chain reaction
PDI: polydispersity index
PEG: polyethylene glycol
PLGA: poly(lactic‐co‐glycolic acid)
qPCR: quantitative polymerase chain reaction
RIPA: radioimmunoprecipitation assay
RNA: ribonucleic acid
SDS/PAGE: sodium dodecyl sulfate/polyacrylamide gel electrophoresis
SEM: scanning electron microscope
TEM: transmission electron microscope
TPGS: D‐ɑ‐tocopheryl polyethylene glycol succinate
TRAP: telomeric repeat amplification protocol

The 37 kDa laminin receptor precursor/67 kDa High Affinity Laminin Receptor (LRP/LR) is a highly conserved nonintegrin transmembrane receptor, which is able to bind a variety of molecules such as prion proteins, elastin and laminin, and is implicated in a variety of disorders [1, 2, 3]. Although it performs its major functions on the cell surface, LRP/LR is additionally found in the cytosolic domain and the perinuclear compartment, where it plays a role in many additional processes, such as cell proliferation and growth [1, 4], nuclear structure maintenance as well as translational functions [5].

It has further been established that LRP/LR plays a key role in mediating telomerase activity [4, 6, 7, 8], whereby LRP/LR increases telomerase activity to promote cell viability and prevent cellular senescence. Further evidence shows that the overexpression of LRP::FLAG increases the telomerase catalytic subunit (hTERT) protein levels as well as telomerase activity, and subsequently telomere length within MRC5 and HEK‐293 cell lines. This may suggest that LRP/LR aids in the formation of the active telomerase enzyme from its major hTERT and hTERC components. This is evidenced by the co‐localisation between LRP and hTERT, as well as the increase in telomerase activity observed with increased LRP levels [6, 9, 10, 11]. Interestingly, LRP::FLAG overexpression has been shown to have a positive effect on the Alzheimer's disease state, where the increased LRP levels resulted in decreased phosphorylated Tau levels, Aβ shedding and concomitantly, intracellular Aβ levels [10, 11]. This interaction therefore has potential applications for the treatment of various diseases, including age‐related disorders and neurodegenerative diseases. However, using the LRP::FLAG plasmid‐based transfection method, as in our previous studies, has limited clinical applicability *in vivo*, due to likely off‐target effects from the gene‐modifying transfection process. Therefore, this study included optimising the production, and use, of poly(lactic‐co‐glycolic acid) (PLGA) nanocapsules to encapsulate isolated LRP protein, and thereafter assess the efficacy in a cell model.

In this context, biodegradable polymeric PLGA nanoparticles in the form of hollow nanocapsules were used, to serve as nanocarriers of macromolecular drugs, such as the LRP protein. These provided a promising alternative due to their advantages over regular drug delivery systems such as plasmid transfection and viral vectors [12, 13]. PLGA is FDA approved and has been used as a delivery system for various applications such as cancer (targeted chemotherapeutic delivery system), diabetes (insulin carrier), bacterial infections (antibiotics), vaccines and dissolvable stitches [14, 15]. Therefore, PLGA nanocapsules can be exploited as a drug delivery vehicle.

The development of a PLGA encapsulated LRP protein‐based drug could therefore be very beneficial, since the nanocapsule conveys protection and would facilitate controlled uptake of the LRP protein into cells, without the protein being degraded via natural immune responses. Due to the wide range of applications that elevating LRP levels has in the treatment of different disorders, this could represent a safer alternative to using plasmid transfection as a treatment and could potentially be used for the treatment of age‐related diseases, through its ability to increase telomerase activity.

## Materials and methods

### Nanoparticle drug design

#### Synthesis, purification and characterisation of PLGA nanocapsules

PLGA nanocapsules were synthesised using the double emulsification‐solvent evaporation technique [15], with minor changes. PLGA nanocapsules were produced to encapsulate purified LRP protein, as well as empty nanocapsule controls. Briefly, 100 mg of PLGA polymer (Merck, Darmstadt, Germany) was dissolved in 1 mL of either ethyl acetate (EA) (Associate Chemical Enterprise, Southdale, South Africa) or dichloromethane (DCM) overnight to achieve a 100 mg·mL^−1^ initial concentration. This was later increased to 2 mL of EA or DCM for an initial concentration of 50 mg·mL^−1^, as it produced more reliable syntheses, protein encapsulation was therefore performed at this concentration. For protein encapsulation, 200 μL of 5 mg·mL^−1^ purified LRP (1 mg total), in a water suspension, was added to the PLGA solution using a glass Pasteur pipette and repeatedly aspirated to create the first emulsion.

This emulsion (2.2 mL) was then added dropwise to the centre of 4 mL 0.3% (w/v) vitamin E (TPGS) (Merck), while vortexing. This second emulsion was then further vortexed for 15 s and then immediately sonicated on ice for five cycles of 10 s each at 40% amplitude using a 700 W probe sonicator to form nanocapsules by cavitation. This nanocapsule solution was then transferred to 45 mL of 0.3% (w/v) vitamin E (TPGS) and was rapidly stirred for 3 h, to facilitate solvent evaporation and subsequent nanocapsule hardening [15], resulting in the ‘raw’ sample.

The nanocapsules were collected in the supernatant by centrifugation at 500, 8000, 10 000, 12 000 or 14 000 ***g*** for 1 min or by 0.45 μm filtration and further purified by centrifugation at 14 000 ***g***, to remove the vitamin E (TPGS), after which the pellet was resuspended in Rnase/Dnase free dH_2_O. This wash step was performed three times, and the resultant nanoparticle solution was retained for lyophilisation, resulting in the ‘purified’ sample.

To characterise the morphology and size of the nanocapsules, scanning electron microscopy (SEM) was used in addition to dynamic light scattering (DLS). To prepare the samples for characterisation by SEM, 10 μL of unpurified nanocapsules, nanocapsules isolated at 8000, 10 000, 12 000, 14 000 and 500 ***g*** as well as 0.45 μm filtered nanocapsules were placed on individual strips of double‐sided carbon tape on stainless‐steel specimen stubs and allowed to dry overnight in a laminar flow hood. The stubs containing the samples were subsequently sputter coated with a single coat of gold–palladium and one coat of carbon, each for 30–120 s. The nanoparticles were subsequently imaged using the FEI Spirit 120 kV transmission electron microscope (TEM), at as well as the FEI Quanta 400 FEG‐SEM at the Wits University, Microscopy and Microanalysis Unit (MMU) (high voltage‐HV) or the TESCAN MIRA3 FEG‐SEM at the University of Cape Town, Imaging and Analysis Centre (IAC). Particles were visualised under a beam strength of 2 (MIRA3) – 30 kV (Quanta, Field Electron and Ion Company ‐ Hillsboro, OR, USA), at a working distance of 5–15 nm and a spot size of 1. A minimum of three images were captured per batch of nanoparticles to attain a representative sample of the size and morphology of the particles. DLS was further performed to measure the hydrodynamic size of the particles and the polydispersity index (PDI) of the ‘raw’ samples, as well as the unpurified samples centrifuged at 500 ***g*** (still in vitamin E (TPGS)) and the purified samples (removed vitamin E (TPGS) and resuspended in Dnase/Rnase‐free water) using a zetasizer nano zs‐90 (Malvern Panalytical, Worcestershire, UK).

#### Micro‐BCA assay

BCA assays (Thermo Fisher Scientific, Waltham, MA, USA) were performed to confirm successful protein encapsulation. Empty and LRP‐containing lyophilised nanocapsules were resuspended in radioimmunoprecipitation assay (RIPA) buffer (Merck) or dimethyl sulfoxide (DMSO) at a concentration of approximately 65 mg·mL^−1^ for 10 min to disrupt the polymer shell. These samples were then incubated in the BCA working solution for 2 h at 37 °C until a colour change was achieved. The absorbance was measured at 562 nm using a VICTOR^®^ Nivo™ Multimode Microplate Reader (PerkinElmer, Shelton, CT, USA). A BSA standard curve was constructed to extrapolate the amount of protein present.

### Cell culture protocol

Human embryonic kidney (HEK‐293) cells (ATCC, Manassas, VA, USA) [16] were used to assess the cytotoxicity of the empty nanocapsules, as well as to assess the efficacy of using the PLGA encapsulated LRP nanocapsules as a treatment. These cells are known to display relatively high levels of TERT and telomerase activity and have previously been used to optimise procedures involving telomerase activity and TERT expression [17].

HEK‐293 cells were cultured in Dulbecco's Modified Eagle's Medium (DMEM) (Hyclone, Logan, UT, USA) supplemented with 10% fetal bovine serum (FBS) (Biowest, Nuaillé, France) and 1% Penicillin‐Streptomycin (Thermo Fisher Scientific). The cells were kept in a 5% CO_2_, 37 °C humidified atmosphere.

### MTT cell viability assay

The MTT (3‐(4,5‐dimethylthiazol‐2‐yl)‐2,5‐diphenyltetrazolium bromide) (Thermo Fisher Scientific) assay was used to determine cell viability of the cells treated with both empty and encapsulated LRP nanocapsules. HEK‐293 cells were seeded into a 96‐well plate at 5000 cells per well and allowed to attach overnight, after which the cells were treated with 1%, 5% and 10% (v/v) empty and LRP encapsulated within PLGA nanocapsules and incubated under normal conditions for 48 h. MTT solution was then added to the cells at 0.5 mg·mL^−1^ final concentration and incubated for 4 h. The resulting formazan crystals were solubilised with DMSO (Associate Chemical Enterprise), and the absorbance measured at 570 nm using a spectrophotometer (VICTOR^®^ Nivo™ Multimode Microplate Reader; PerkinElmer). Each treatment and control were performed in biological and technical triplicates. The controls included the no‐cell control, no‐MTT control, cell death positive control (treated with 1% triton x‐100; Bio‐Rad, Hercules, CA, USA) and the untreated control. The percentage cell viability was then established by calculating the treated values as a percentage of the corresponding untreated values for each biological repeat, with the untreated set to 100%.

### Western blot

After the HEK‐293 cells were treated with both the empty and PLGA encapsulated LRP nanocapsules (1% and 5%), western blot analysis was performed to determine whether the LRP was successfully delivered to the cells.

Cell pellets were incubated in 200 μL 1× RIPA buffer for 20 min to lyse the cells. The protein extract was isolated, and the concentration of each sample was then standardised using the BCA assay. Protein extracts were resolved on a 10% Sodium dodecyl sulfate/polyacrylamide gel electrophoresis (SDS/PAGE) gel at 120 V in 1× electrophoresis tank buffer (25 mm Tris, 192 mm glycine; Merck).

The proteins were electro‐transferred from the gel to the membrane in the presence of a 1× transfer buffer (25 mm Tris, 192 mm glycine, 20% (v/v) methanol; Associated Chemical Enterprise) using the Trans‐Blot^®^ Turbo™ Transfer System (Bio‐Rad). Membranes were thereafter blocked for 1 h using 5% BSA in PBST (1× PBS and 0.1% Tween 20). After blocking, primary antibody (1 : 10 000 primary labelled β‐actin and Hu IgG1‐iS18) diluted in 3% BSA in 1X PBST was added to the blots and incubated overnight with gentle shaking at 4 °C. The membranes were subsequently washed in 1× PBST five times, for 5 min each, after which the respective membranes were incubated in secondary antibody (anti‐human IgG‐HRP) for 1 h (in the dark, at room temperature), followed by five washes. Finally, the membranes were incubated in Clarity™ Western ECL Blotting Substrate (Bio‐Rad) and visualised using the ChemiDoc™ Imaging System (Bio‐Rad). Densitometric analysis was further performed with the image lab (v5.1) software, whereby all values were further normalised against the β‐actin loading control.

### Telomerase activity

Telomerase activity was assessed using a modification of the Telomeric Repeat Amplification Protocol ([18]). Briefly, RNA and protein were extracted in 200 μL of 3‐((3‐cholamidopropyl) dimethylammonio)‐1‐propanesulfonate (CHAPS) lysis buffer, on ice for 30 min, after which the samples were centrifuged at 12 000 ***g*** for 20 min at 4 °C, and the supernatant snap frozen. Subsequently, the protein for all experimental and control reactions was quantified with the NanoDrop™ One (Thermo Fisher Scientific) and standardised to 500 ng·μL^−1^. In a 96‐well plate, the TRAP reaction mixture and 2 μL of the standardised protein samples solution, for a total of 1 μg, were loaded into each well to a final volume of 12.5 μL. This reaction Mastermix consisted of Luna® Universal qPCR Mastermix (New England Biolabs, Ipswich, MA, USA), TS and ACX primers (as described by Bignoux et al. [8]), EGTA and PCR‐grade water. The CFX96™ Touch qPCR System (Bio‐Rad) was employed to perform qPCR analysis on all samples, using the following cycling parameters: One cycle of 30 min at 37 °C (telomere extension step), and 2 min at 95 °C, followed by 45 cycles of amplification with denaturation at 95 °C for 15 s, annealing at 59 °C for 60 s, and extension at 45 °C for 10 s. Minus telomerase (consisting only of CHAPS lysis buffer), no template (PCR‐grade water) and heat‐treated (incubated at 90 °C for 10 min to inactivate any telomerase activity) negative controls were included. A telomerase positive cell extract was included as a positive control. The Bio‐Rad cfx maestro software was used to analyse the Cq values, and all values were normalised as a percentage of the untreated controls (set to 100%), after background subtraction of the heat‐treated samples.

### Data analysis and statistical evaluation

Data analysis was performed in Microsoft Excel 365 Pro (Microsoft Corporation, Redmond, WA, USA), whereas statistical analysis was performed in graphpad prism (v6.05). A one‐way ANOVA analysis was performed where relevant, using Dunnets and Newman–Keuls multiple comparisons *post hoc* analysis, followed by pairwise comparisons using a two‐tailed student's *t*‐test, both at a 95% confidence interval, where *P* values < 0.05 were considered statistically significant (*P < 0.05, **P < 0.01 and ***P < 0.001).

## Results and Discussion

The overexpression of LRP has been shown to have therapeutic effects in ageing and neurodegenerative disease models [6, 10, 11]. In order to translate this research to *in vivo* models, we developed an LRP protein‐based drug using PLGA nanocapsules as the drug delivery system. Here, PLGA was used as it is biocompatible, biodegradable and nontoxic, theoretically leading to a slower, longer lasting release of the therapeutic LRP. Additionally, the elevation of LRP levels in this manner should not adversely affect cell viability as LRP is a natural protein found in the body.

### Optimisation of PLGA nanocapsule synthesis

**Fig. 1:**
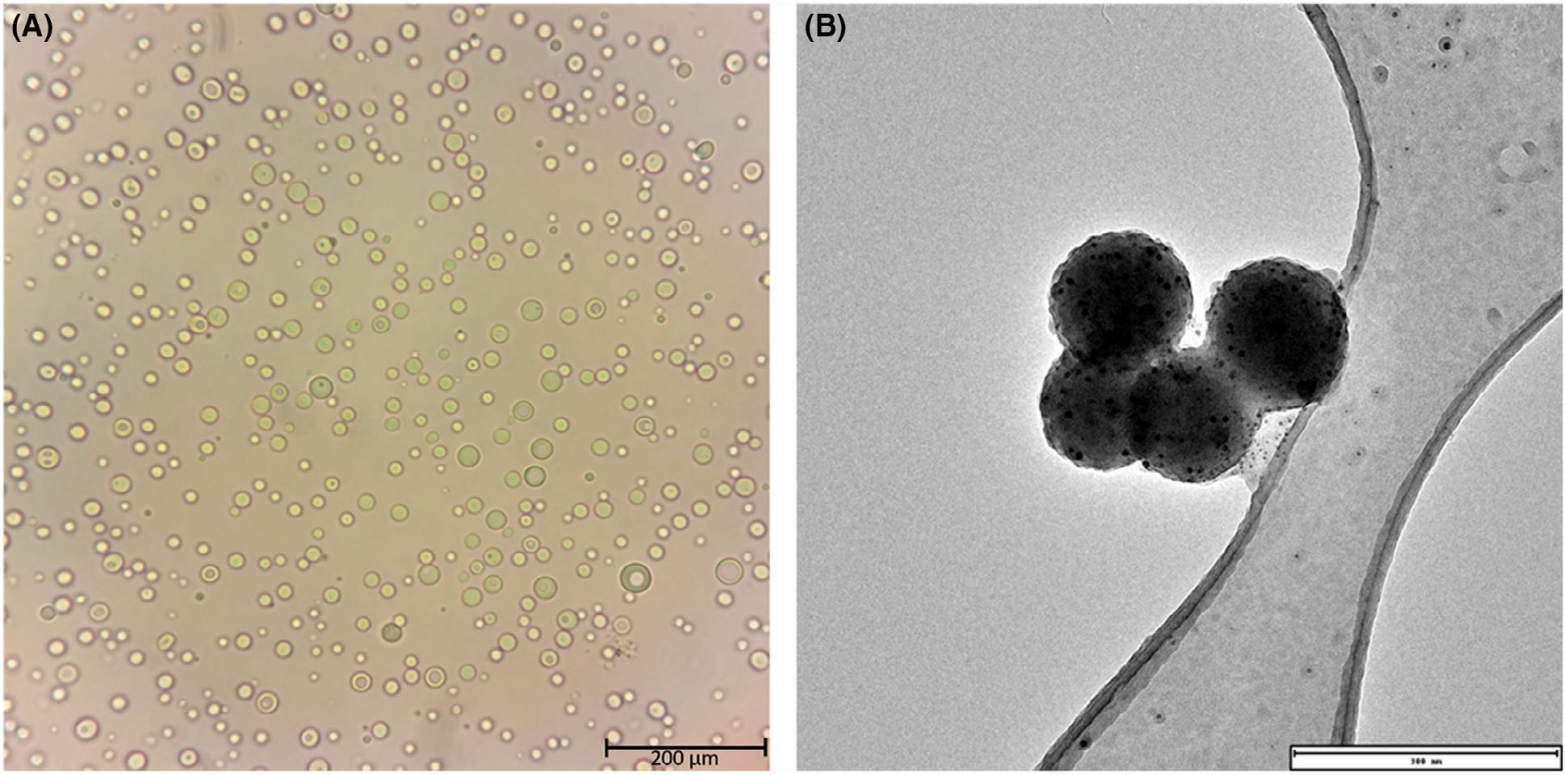
Light microscope and transmission electron microscope (TEM) images of poly(lactic‐co‐glycolic acid) (PLGA) nanocapsules. (A) A light micrograph (100× magnification; Zeiss Primovert) of hollow PLGA microcapsules. These microcapsules represent the larger particles that were removed during the purification process (pelleted out by centrifugation at 500 ***g***) and confirm spherical nanoparticles were formed. Scale bars represents 200 μm. (B) A TEM micrograph (FEI Spirit 120 kV TEM; Wits Microscopy and Microanalysis Unit) of the small fraction (supernatant) of PLGA nanoparticles. This confirmed that the PLGA nanoparticles were within the desired size range of 200–450 nm in diameter. *n* = 3; scale bar represents 300 nm.

PLGA nanocapsule synthesis was optimised using the double emulsion, solvent evaporation method [15] with EA or DCM as the solvent, and vitamin E (TPGS) as the emulsifier. After each synthesis, the nanocapsules were characterised using a combination of TEM, SEM and DLS. Images were initially taken of the large fraction (pelleted by centrifugation at 500 ***g***) using light microscopy (Fig. 1A) to determine whether the nanocapsule synthesis method yielded spherical particles prior to performing electron microscopy. This was done as an initial observation before preparing the samples derived from the supernatant for electron microscopy. It was found that indeed the synthesis method produced spherical nanoparticles. Furthermore, the supernatant was then imaged (using TEM), to determine the size of the isolated nanoparticles, and it was confirmed these nanoparticles were within the 200–500 nm size range (Fig. 1B).

The PLGA polymer can be solubilised in a variety of solvents, including DCM and EA [15]. These compounds are water‐immiscible and can dissolve the polymer in preparation for emulsion [19]. Initially, each solvent was tested under the same conditions to evaluate the resulting nanocapsules. Both EA and DCM were used to successfully synthesise PLGA nanocapsules, using an initial PLGA concentration of 100 mg·mL^−1^.

**Fig. 2:**
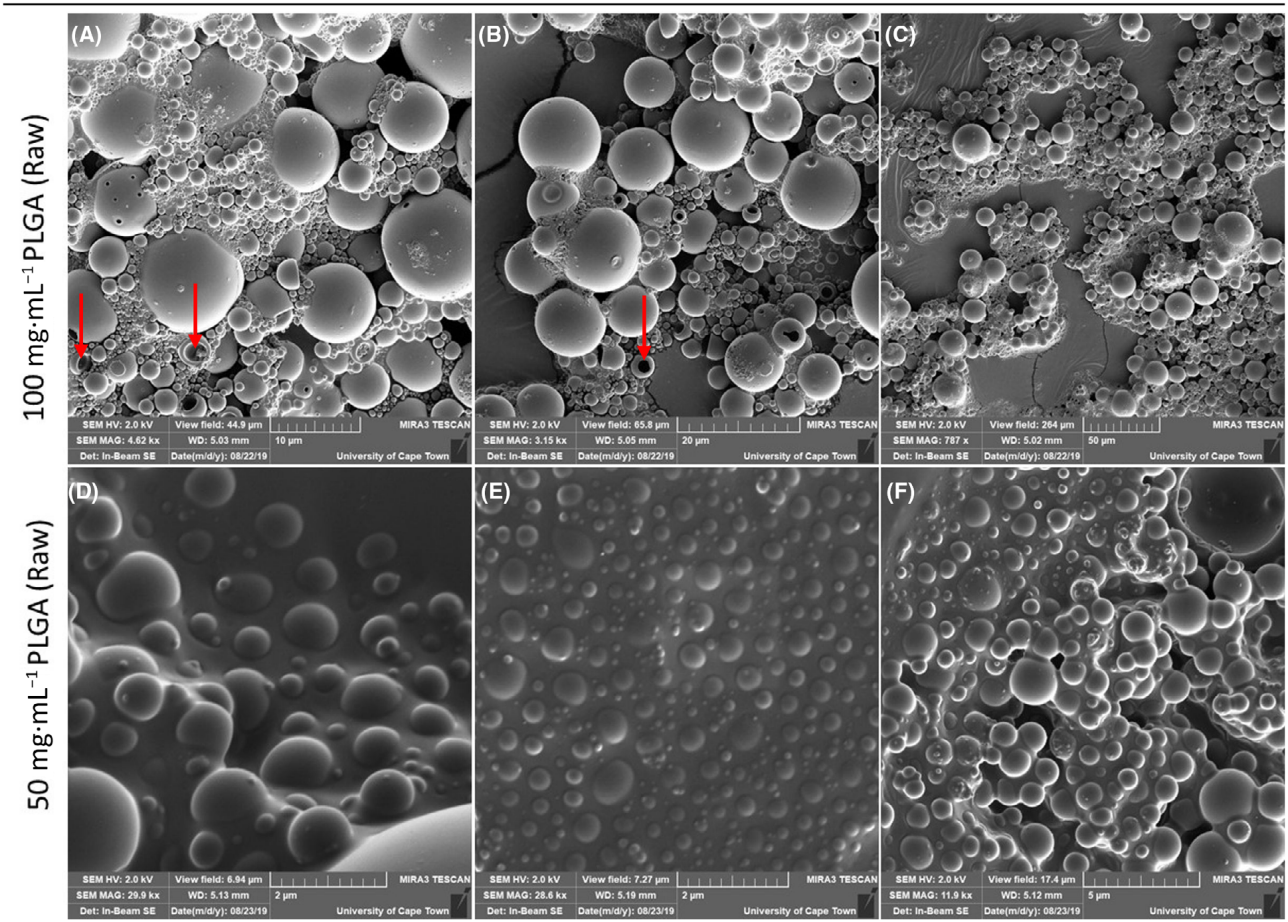
Effect of initial poly(lactic‐co‐glycolic acid) (PLGA) (in solvent) concentration on nanocapsule polydispersity. Scanning electron microscope (SEM) micrographs depicting unpurified (raw) PLGA nanocapsules, synthesised using dichloromethane (DCM) and vitamin E (TPGS) at both 50 and 100 mg·mL^−1^ initial concentration, (gold–palladium coated; TESCAN MIRA3 FEG‐SEM, University of Cape Town CIA). Three micrographs were taken per sample to represent the size distribution more accurately. (A–C) Represent 100 mg·mL^−1^ initial polymer concentration (*n* = 3). Scale bars represent 10, 20 and 50 μm, respectively. (D–F) represent 50 mg·mL^−1^ initial polymer concentration (*n* = 3). Scale bars represent 2, 2 and 5 μm, respectively. Through the observation of damaged nanoparticles, it was confirmed that hollow nanocapsules were successfully synthesised (red arrows). Images show that the higher initial PLGA concentration (100 mg·mL^−1^) resulted in a larger nanocapsule size distribution than that of the 50 mg·mL^−1^ initial PLGA concentration.

After the PLGA was dissolved in the primary solvent (DCM or EA), the resulting solution was rapidly added to a vitamin E (TPGS) solution. It was theorised that decreasing the viscosity of the solution, by doubling the solvent volume, while keeping the amount of PLGA polymer consistent, would lead to more rapid and consistent synthesis and nanoparticle size distribution. It was postulated that the initial concentration would influence the uniformity of the synthesised nanocapsules, due to the dropwise addition of the solubilised PLGA. It was expected that a higher concentration would not be efficiently sonicated, leading to increased polydispersity. In fact, there seems to be a consensus that the viscosity of the initial polymer/solvent solution is vital for the formation of low‐polydisperse PLGA nanocapsules [20, 21]. Therefore, both a 50 and 100 mg·mL^−1^ initial PLGA (in DCM or EA) concentration were tested. Indeed, it was found that the nanocapsules produced using the 50 mg·mL^−1^ initial concentration of PLGA exhibited less size variation and smaller nanocapsules overall (Fig. 2D–F) in comparison with those produced using a 100 mg·mL^−1^ initial dilution of PLGA (Fig. 2A–C). Furthermore, the nanoparticles were hollow, and thus, we confirmed that nanocapsules were being produced. Since no morphological difference could be seen between nanocapsules synthesised using DCM and EA and that there was some evidence to suggest that DCM can lead to protein aggregation [22], as a precaution, the 50 mg·mL^−1^ initial dilution of PLGA in EA was used for all subsequent syntheses.

### Purification of PLGA nanocapsules

**Fig. 3:**
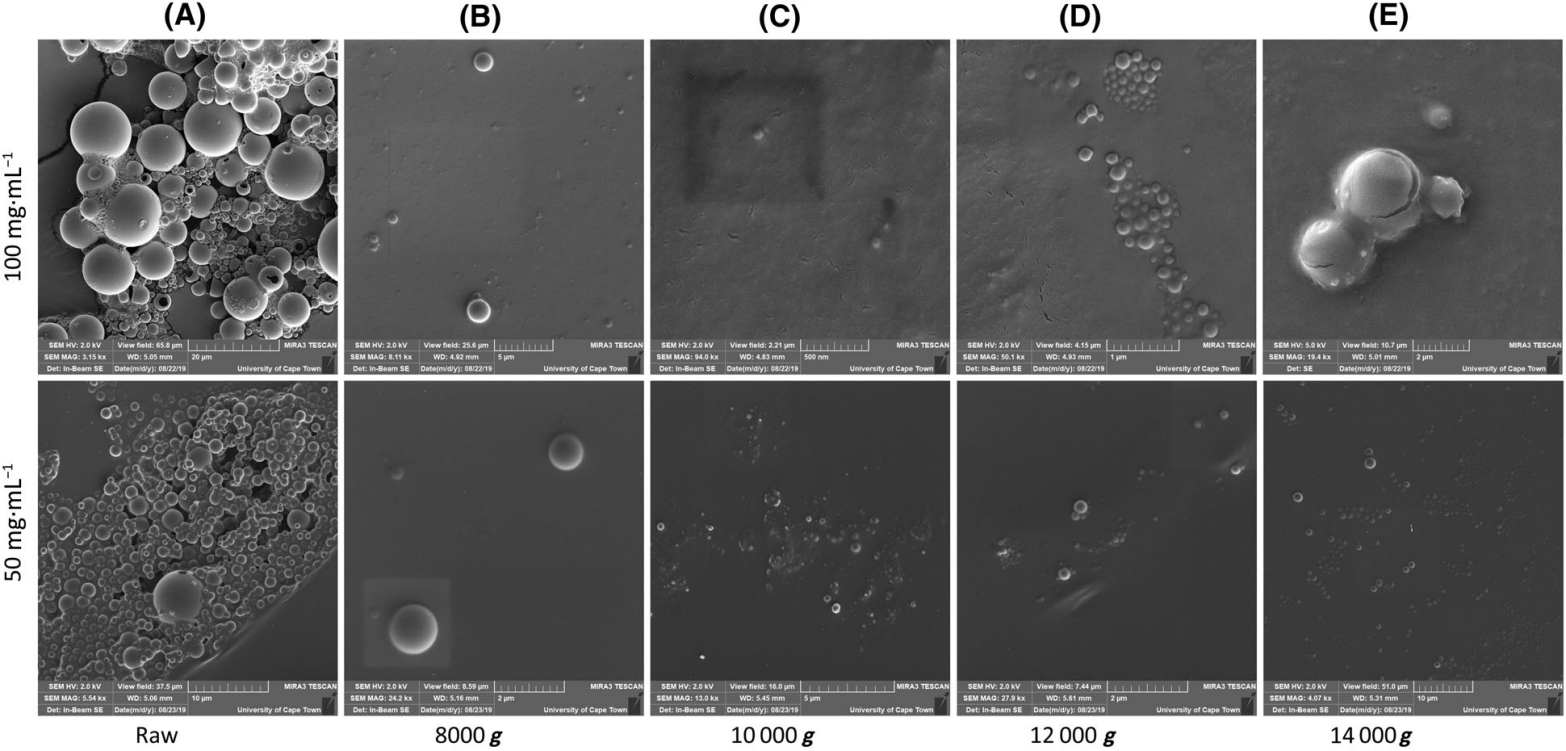
Poly(lactic‐co‐glycolic acid) (PLGA) nanocapsules purification by centrifugation at high centrifugation speeds. Scanning electron microscope (SEM) micrographs depicting high centrifugation speed isolated PLGA nanocapsules and indicating the large sample loss. PLGA nanocapsules were synthesised using dichloromethane (DCM) and vitamin E (TPGS) at both 50 and 100 mg·mL^−1^ initial polymer concentrations. (A) unpurified nanocapsules (*n* = 3, scale bars represent 20 and 10 μm), (B) nanocapsules isolated at 8000 ***g*** (*n* = 3, scale bars represent 5 and 2 μm), (C) nanocapsules isolated at 10 000 ***g*** (*n* = 3, scale bars represent 500 nm and 5 μm), (D) nanocapsules isolated at 12 000 ***g*** (*n* = 3, scale bars represent 1 and 2 μm), (E) nanocapsules isolated at 14 000 ***g*** (*n* = 3, scale bars represent 2 and 10 μm), (gold–palladium coated; TESCAN MIRA3 FEG‐SEM, University of Cape Town CIA). The supernatant of the high centrifugation samples showed very few nanocapsules. Due to this significant loss, high centrifugation could not be used as an isolation method.

After the optimisation of the nanocapsule synthesis, various centrifugation parameters (8000, 10 000, 12 000, 14 000 ***g***) were tested in attempt to isolate and purify nanocapsules of the desired size (under 450 nm). These centrifugation parameters were chosen to remove larger particles found in the subsequent pellet and retain smaller nanoparticles within the supernatant. Due to the nanometre size range of the particles, it was postulated that the high centrifugation speeds would still retain the desired nanocapsules while removing the vast majority of larger particles. Two batches (50 and 100 mg·mL^−1^ initial PLGA [in solvent] concentration) of PLGA nanocapsules were aliquoted into multiple vials, each of which were then used to test the various centrifugation parameters. The resulting supernatant of each sample was visualised using SEM, and although the desired PLGA nanocapsule size range was successfully isolated using the high centrifugation parameters, there was a significant loss of nanoparticles (Fig. 3).

**Fig. 4:**
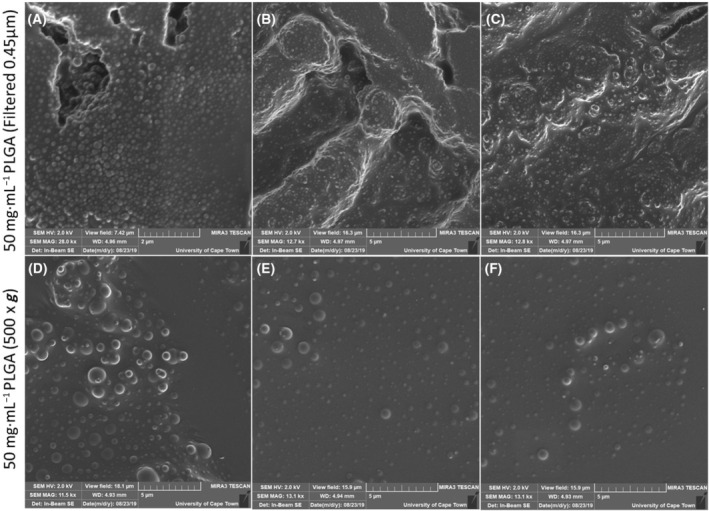
Poly(lactic‐co‐glycolic acid) (PLGA) nanocapsules purification by 0.45 μm filtration and centrifugation at 500 ***g***. Scanning electron microscope (SEM) micrographs depicting 0.45 μm filtered (A–C) (*n* = 3, Scale bars represent 2, 5 and 5 μm) and 500 ***g*** isolated (D–F) (*n* = 3, Scale bars represent 5 μm) PLGA nanocapsules, synthesised using ethyl acetate (EA) and vitamin E (TPGS) at 50 mg·mL^−1^ initial concentration, (gold–palladium coated; TESCAN MIRA3 FEG‐SEM, University of Cape Town CIA). Three micrographs were taken per sample to represent the size distribution more accurately. Both filtration and 500 ***g*** isolation resulted in a narrow nanocapsule size distribution.

Subsequently, low centrifugation isolation (500 ***g*** for 1 min) and 0.45 μm filtration was attempted. Both methods successfully removed large nanocapsules and isolated the desired smaller nanocapsules from the solution (Fig. 4). It was evident that both techniques showed significantly more uniform nanocapsule size distribution than was seen with the high centrifugation particle isolation techniques. The overall yield for both techniques was also substantially improved over the previous isolation attempts. The filtered sample (Fig. 4A–C), however, showed a reduced yield compared with the 500 ***g*** isolated sample (Fig. 4D,E). This was confirmed by the reduced opacity of the nanoparticle solution, the smaller pellet size after the purification step as well as reduced number of observable nanoparticles under SEM. This was due to the filters rapidly becoming clogged, which prevented smaller nanoparticles from passing through. Additionally, despite having a very uniform nanoparticle size distribution, the filtered sample contained far smaller nanocapsules (±200 nm in diameter) than that of the 500 ***g*** isolated sample, which is in the lower end of the 200–500 nm desired size range. Interestingly, the size of the PLGA nanocapsules has been found to be a significant contributor to the bioavailability and distribution of the therapeutic construct. It has been found that smaller nanoparticles are more likely to be rapidly removed by the body through the kidneys [23], whereas larger particles (> 200 nm in diameter) can become more concentrated in the spleen, liver, bone marrow and lungs [24]. Medium‐sized nanoparticles (±100 nm in diameter), on the contrary, have been found to circulate in the blood for longer periods than larger nanoparticles, as they do not get trapped in these organs as easily. However, the smaller nanoparticles (< 100 nm) can have a higher initial burst release of the therapeutic compound, which can have cytotoxic effects [23, 24, 25]. Furthermore, nanoparticles in the 250–500 nm size range allow for the administration of the particles, without risking the blockage of blood vessels. Accordingly, this size range allows for the particles to be delivered orally, rectally, transdermally, ocularly, nasally, subcutaneously, intraperitoneally, intramuscularly and directly into the systemic circulatory system [26, 27]. Therefore, without an active targeting approach using surface modifications, PLGA nanoparticles can be made to passively target relatively specific sets of organs or be designed for prolonged release through size alone. For this reason, the size and morphology of the nanocapsules would need to be strictly controlled. Thus, the optimal size range decided on for this study was 200–500 nm, to allow for passive targeting and prolonged release. Due to these factors, the 500 ***g*** isolation technique was used for future experiments.

**Fig. 5:**
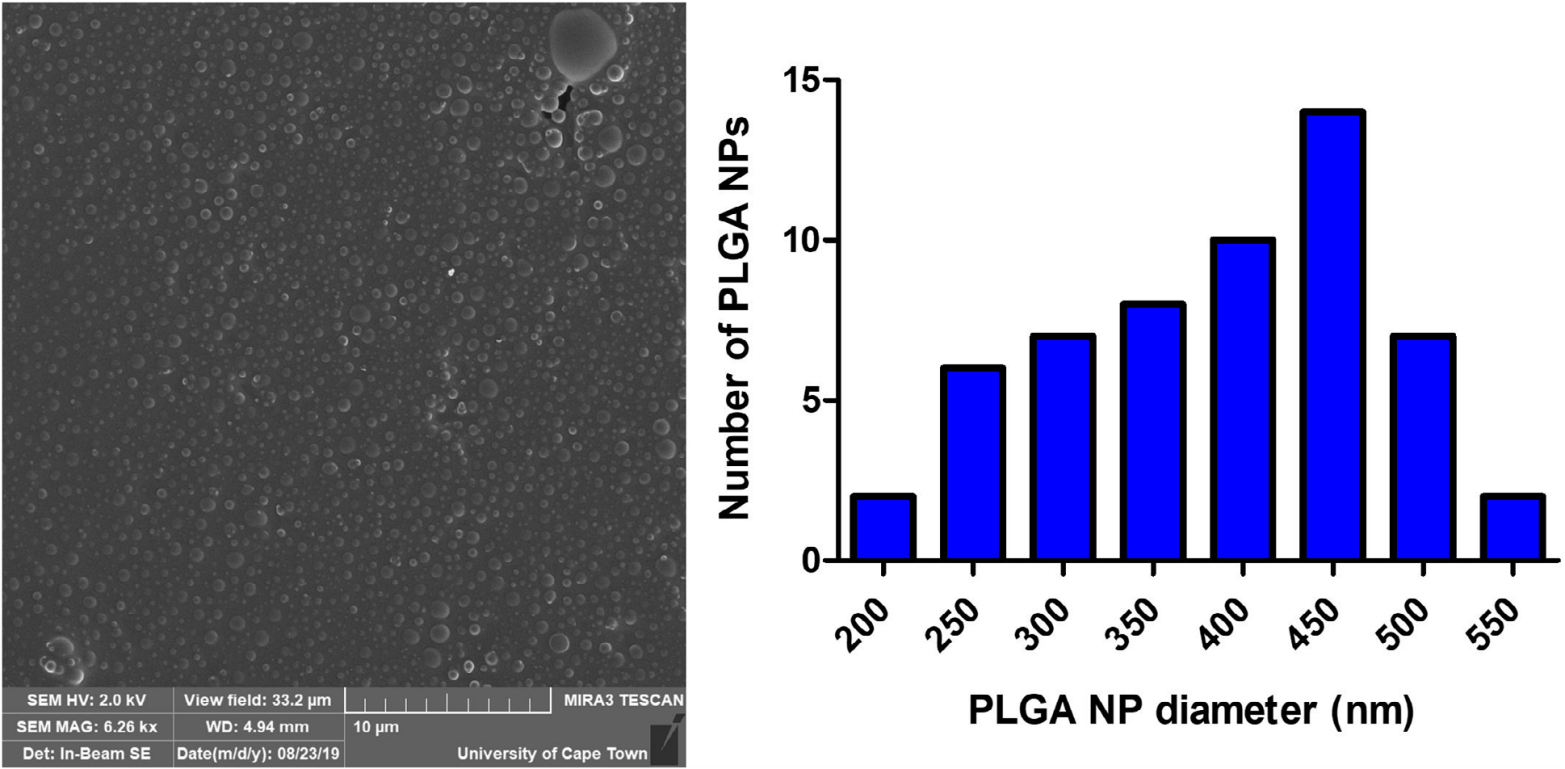
Evaluation of the size distribution of poly(lactic‐co‐glycolic acid) (PLGA) nanocapsules. Scanning electron microscope (SEM) micrograph (left) of 500 ***g*** isolated PLGA nanocapsules, synthesised using ethyl acetate (EA) and vitamin E (TPGS) at 50 mg·mL^−1^ initial concentration (gold–palladium coated; TESCAN MIRA3 FEG‐SEM, University of Cape Town CIA) was analysed using imagej (National Institutes of Health and LOCI, University of Wisconsin) to determine the size distribution (right). The average diameter was determined to be 390 nm. *n* = 3; scale bar represents 10 μm.

Subsequently, the average diameter and the polydispersity index (PDI) of the PLGA nanocapsules was determined by DLS (Table 1). The PDI describes the uniformity of particle size distribution, where values greater than 0.7 indicate a broad size distribution and values below 0.05 are considered monodisperse. Isolated, purified (resuspended in RNase‐free water) nanoparticles were compared with isolated nonpurified and raw nanoparticle samples. The raw nanoparticle sample had an average diameter of 324.87 nm and a PDI of 0.71, whereas the 500 ***g*** isolated, nonpurified sample had an average diameter of 200.27 nm and a PDI of 0.79. This suggests that the isolation procedure does indeed remove many of the larger nanoparticles present in the solution, however with a PDI of over 0.7, these solutions were still considered polydisperse. However, the average diameter of the isolated and purified samples increased to 502.8 nm and the PDI decreased to 0.38. This improvement in the PDI suggests that the purification steps to remove the vitamin E (TPGS) further reduces the size distribution of the sample and is most likely due to the additional high speed centrifugation steps. This means that the initial synthesis (raw sample) and a subsequent 500 ***g*** isolation, followed by a purification step, significantly improved the polydispersity of the nanoparticle solution. Additionally, a size distribution analysis of the isolated and purified PLGA nanocapsule sample was performed on the SEM images using imagej software (National Institutes of Health and LOCI, University of Wisconsin) (Fig. 5). The average diameter was found to be 390 nm with a standard deviation of 88.5 nm. This size disparity between the average diameter obtained by DLS (502.8 nm) and that obtained by SEM is explained by the fact that DLS measures hydrodynamic size, whereas SEM closely represents the actual size of the nanocapsules. This therefore confirmed the nanoparticles produced and purified in this way were indeed within the desired size range of 200–500 nm.

**Table 1:** Dynamic light scattering (DLS) results of poly(lactic‐co‐glycolic acid) (PLGA) nanocapsules synthesised using ethyl acetate.

PLGA sample	Average diameter (nm)	Polydispersity index
Raw Vit‐E TPGS	324.87	0.71
500 ***g*** Vit‐E TPGS	200.27	0.79
500 ***g*** purified	502.80	0.38

### The effects of empty PLGA nanocapsules on cell viability

**Fig. 6:**
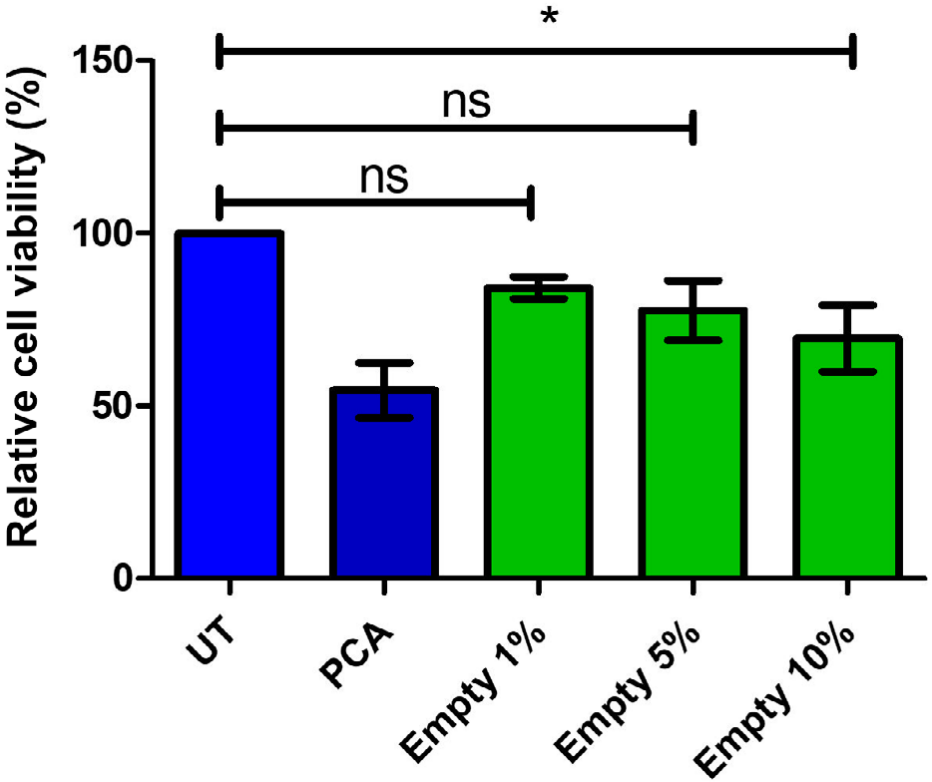
HEK‐293 cell viability after poly(lactic‐co‐glycolic acid) (PLGA) nanocapsule treatment. HEK‐293 cells were treated with 1%, 5% and 10% (v/v) by volume of empty PLGA nanocapsules. Although there was some loss of cell viability for the 1% and 5% treatments, it was nonsignificant compared with the untreated control with a mean decrease of 15.77% and 22.34%, respectively. At the highest concentration (10%), there was a significant decrease in cell viability of 30.41% (*P* ≤ 0.05). A one‐way ANOVA analysis was performed; error bars represent standard deviation; *n* = 3 biological replicates; ns *P* > 0.05, *P ≤ 0.05.

Once the optimisation of the synthesis, isolation and purification of the PLGA nanocapsules was completed, the cytotoxicity of the empty nanocapsules was evaluated in HEK‐293 cells by MTT assay (Fig. 6). Cells were treated with 1%, 5% and 10% (v/v) PLGA nanocapsules, which represents percentage nanoparticles to total blood volume (i.e., *in vivo*). There was no significant decrease in cell viability observed in the cells treated with 1% and 5% PLGA, however, a significant decrease of 30.41% was observed in the cells treated with 10% PLGA. At this treatment concentration, however, the number of nanoparticles present visibly clouded the media and settled on the adherent cells. This likely led to a decreased area in which the cells could migrate, and possibly caused contact inhibition, therefore preventing cell proliferation, rather than decreasing cell viability. Interestingly, Derman et al. [28] showed that treating L929 cells (mouse fibroblast) with empty nanoparticles at very high concentrations (0.5 mg·mL^−1^) resulted in a 30% decrease in cell viability. The 10% empty PLGA treatment mirrors this outcome almost exactly. Nevertheless, the 1% and 5% treatments confirm that the empty PLGA nanocapsules have no significant effect, and the 10% treatment only minimally affected cell viability. Altogether, this confirms that the PLGA nanoparticles are nontoxic and can be used as a delivery vehicle for the LRP protein *in vivo*.

### Protein encapsulation

Due to the cost and time required to produce the LRP protein, the optimisation of protein encapsulation was first performed using BSA. These trials were performed to determine whether the addition of the protein would affect the size and morphology of the synthesised nanocapsules. A protein in water suspension was prepared with 1 mg of BSA, which was encapsulated using the optimised double emulsion solvent evaporation method as previously explained. It was seen that, compared with the empty nanocapsule control (Fig. S1A), the encapsulation of BSA (Fig. S1B) did not significantly affect the size and morphology of the resultant nanocapsules. This was likely due to the fact that the size of the encapsulated proteins is far smaller than that of the nanocapsules, with BSA being less than 20 nm in height [29], compared with the average diameter of the nanocapsules being approximately 400 nm.

To confirm successful encapsulation of BSA, a micro‐BCA assay was performed on the extract from these nanoparticles, which were concentrated down to 1 mL, resuspended in water, and disrupted using DMSO. The BSA protein concentration was determined to be 0.882 mg compared with the 1 mg of added BSA (88.2% encapsulation efficiency). This encapsulation efficiency is relatively high, as there is likely to be a small amount lost during the encapsulation process, and an inevitable loss of protein through the isolation and purification steps. In fact, it is comparable with that of Amini et al., 2017 [30] who encapsulated BSA and obtained an encapsulation efficiency of 81–89%. Based on the high encapsulation efficiency observed with the 1 mg BSA, it was decided to use 1 mg LRP protein during subsequent encapsulation of LRP.

**Fig. 7:**
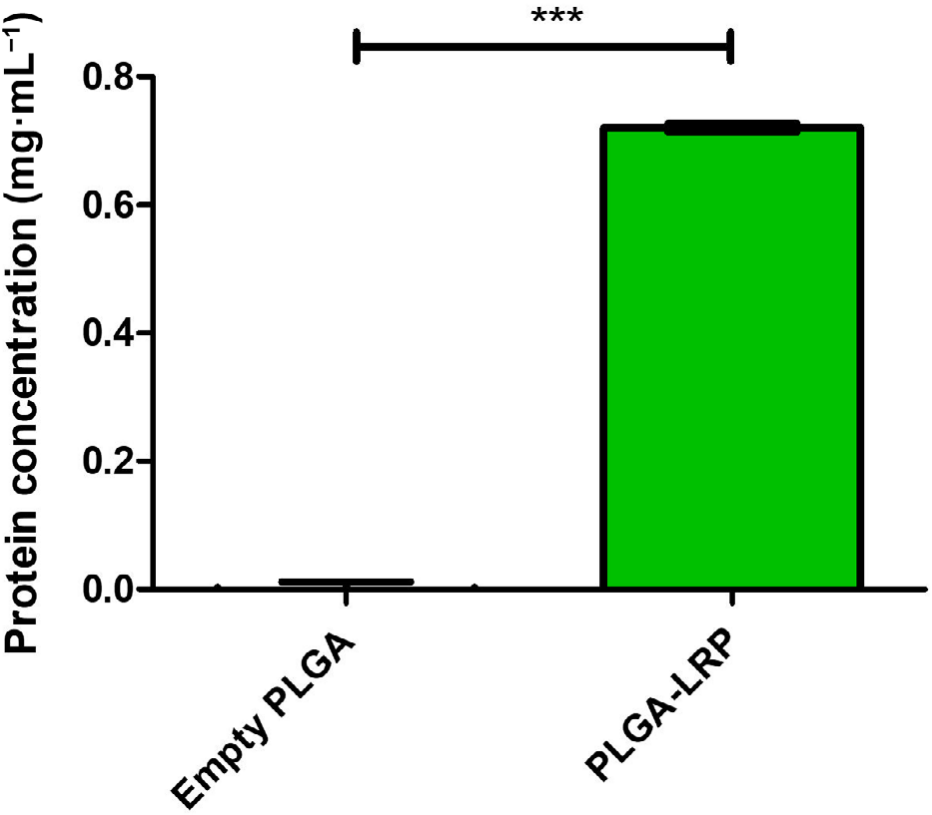
Micro‐bicinchoninic acid (BCA) assay results of empty and encapsulated LRP poly(lactic‐co‐glycolic acid) (PLGA) nanocapsules. Micro‐BCA protein quantification assay was performed on both empty and encapsulated laminin receptor precursor (LRP) PLGA nanocapsules after polymer shell disruption using dimethyl sulfoxide (DMSO). The LRP was detected at a wavelength of 562 nm and the absorbance was significantly higher (*P* ≤ 0.001) than the empty PLGA control. This corresponds to 0.72 mg of LRP, which served as confirmation of LRP encapsulation within PLGA nanocapsules at an encapsulation efficiency of 72%. A one‐way ANOVA analysis was performed; error bars represent standard deviation; *n* = 3 biological replicates; ***P ≤ 0.001.

Synthesised LRP protein (1 mg) was encapsulated in PLGA nanocapsules using the optimised protocol as specified. Synthesis and purification of the LRP protein was performed with assistance from the Protein Structure Function Research Unit (PSFRU) at the University of the Witwatersrand (unpublished results). Multiple encapsulations were performed, and subsequently, a micro‐BCA assay was used to assess the encapsulation efficiency. The micro‐BCA assay showed that there was approximately 0.72 mg LRP present in the concentrated sample out of the initial 1 mg added (Fig. 7), therefore exhibiting a high encapsulation efficiency of 72%, which is comparable to that of the encapsulated BSA trial and previous studies [30].

### Effect of encapsulated LRP treatments on total LRP levels, cell viability and telomerase activity

It has previously been shown that overexpression of LRP causes an increase in cell viability and telomerase activity in various cell culture models, even conveying a rescuing effect against cytotoxic peptides [6, 10, 11]. It has been shown that once LRP/LR has bound to laminin, LRP/LR can then interact with focal adhesion kinase and this interaction then activates the PI3‐kinase/AKT and MEK/ERK1/2 pathways, which are directly related to proliferation and longevity [31]. It has further been established that LRP/LR plays a key role in mediating telomerase activity [4, 6, 7, 8], whereby LRP/LR increases telomerase activity to promote cell viability and prevent cellular senescence, as shown by the reduction in gamma H2AX (which marks sites of DNA damage and DNA breaks) and the senescence biomarker β‐galactosidase [6, 9].

**Fig. 8:**
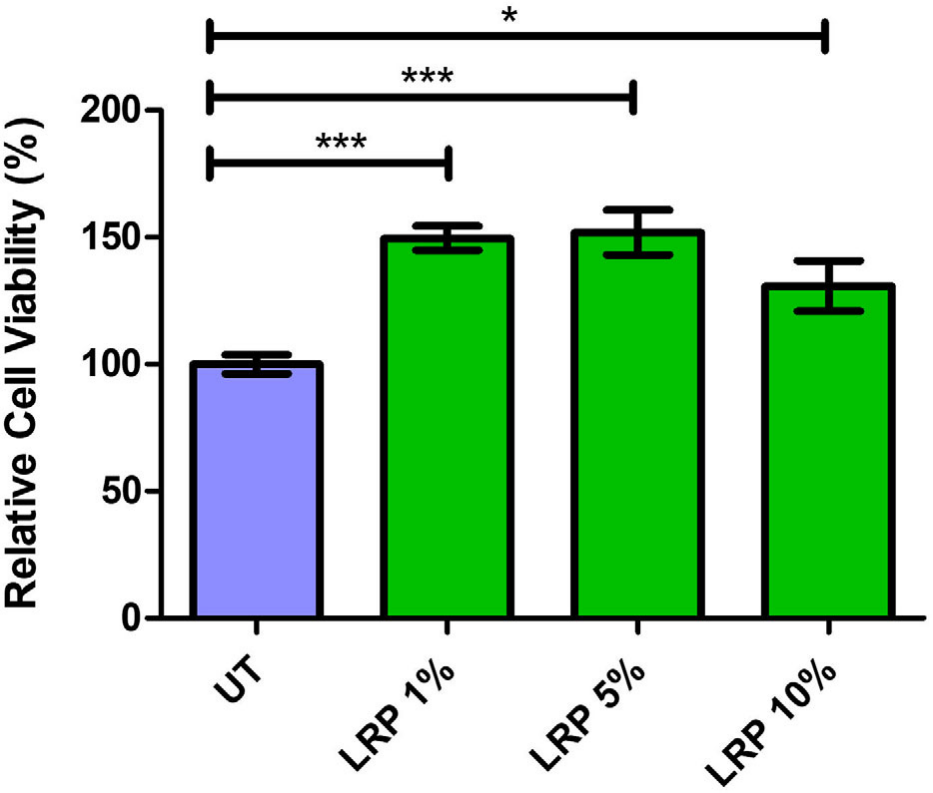
Improved cell viability after laminin receptor precursor (LRP)‐nanoparticle treatment *in vitro*. HEK‐293 cells were treated with 1%, 5% and 10% of both empty and encapsulated LRP poly(lactic‐co‐glycolic acid) (PLGA) nanoparticles and an 3‐(4,5‐dimethylthiazol‐2‐yl)‐2,5‐diphenyltetrazolium bromide (MTT) assay was performed. The 1%, 5% and 10% LRP‐nanoparticle treatments increased cell viability by 49.64% (*P* ≤ 0.001), 51.93% (*P* ≤ 0.001) and 30.82% (*P* ≤ 0.05), respectively. A one‐way ANOVA analysis was performed; error bars represent standard deviation; *n* = 3 biological replicates; *P ≤ 0.05, ***P ≤ 0.001.

To confirm that the LRP encapsulated in the PLGA has a similar effect, HEK‐293 cells were treated with encapsulated LRP nanoparticles at 1%, 5% and 10% (v/v) as previously described, and an MTT cell viability assay was performed. The 1%, 5% and 10% encapsulated LRP treatments significantly improved cell viability of the HEK‐293 cells by 49.64% (*P* < 0.0001), 51.93% (*P* < 0.0001) and 30.82% (*P* = 0.0105), respectively (Fig. 8). This was a very promising result, as it corroborates previous observations where upregulation of LRP::FLAG levels improved cell viability [11]. LRP/LR is theorised to increase cell proliferation and survival by maintaining chromosome stability through the Midkine heparin‐binding growth factor, which leads to the reduction in apoptosis and an increase in cell survival markers [32]. This would suggest that the treatment has similar effects to that of the upregulation.

Interestingly, the cells receiving the 10% treatment showed a significant increase in cell viability, despite the reduction in viability observed after treatment with empty nanocapsules at this concentration. This suggests that the LRP protein is able to rescue the cells from the cytotoxic effect of the PLGA, providing further evidence that the LRP protein was successfully delivered to the cell.

**Fig. 9:**
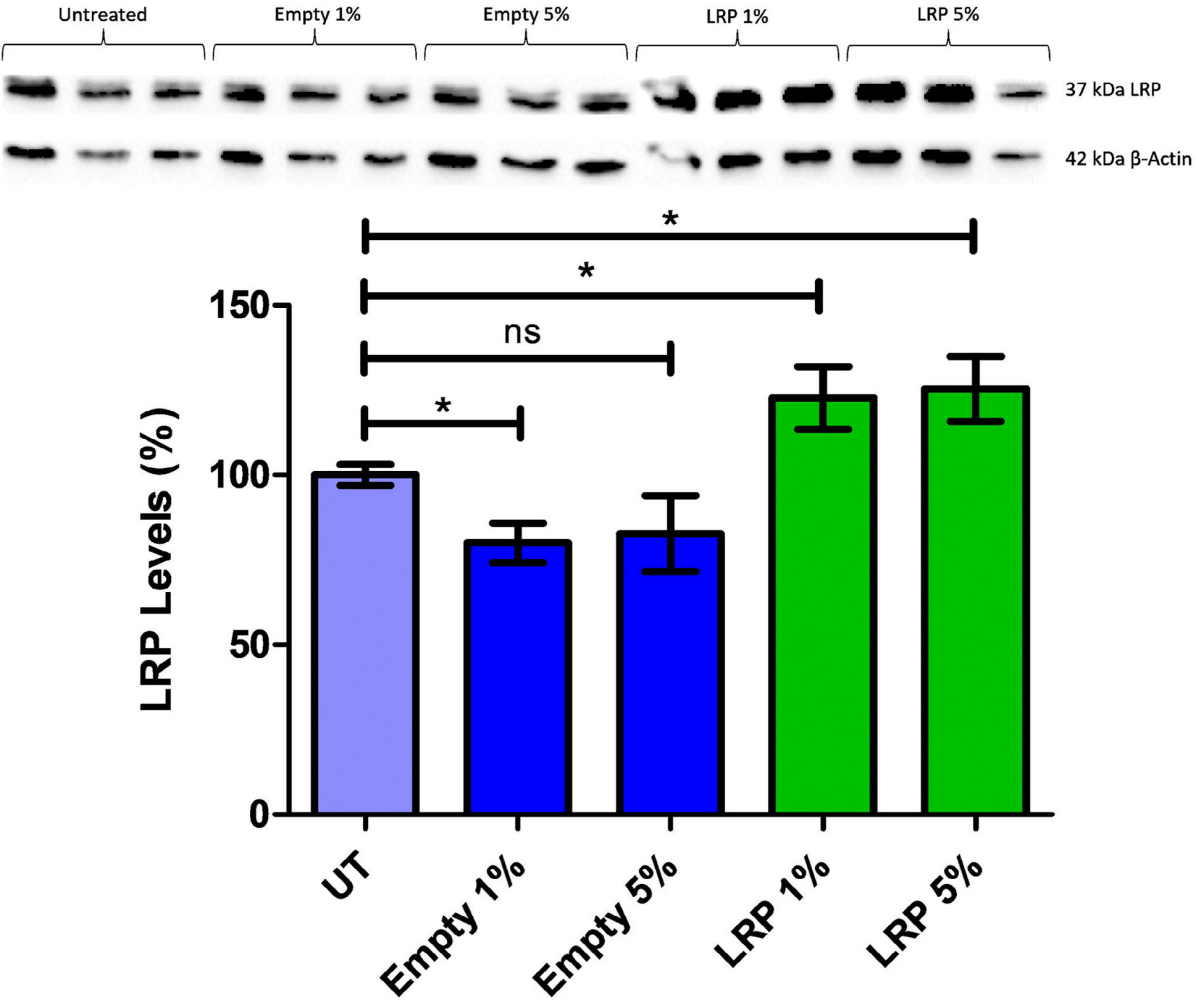
Western blot analysis of encapsulated laminin receptor precursor (LRP) poly(lactic‐co‐glycolic acid) (PLGA) nanoparticle treatments on HEK‐293 cells. Western blots were performed to determine the total LRP protein levels in both untreated and PLGA nanocapsule‐treated HEK‐293 cells. The band density of the 37 kDa LRP was compared with a 42 kDa β‐actin control using Image Lab 4.0; Bio‐Rad. LRP levels of the treated samples where then compared with the untreated control (set to 100%). A significant 20% decrease in LRP levels was detected in the 1% empty nanocapsule treatment (*P* ≤ 0.05) and a significant increase was detected for both the 1% and 5% encapsulated LRP treatments (22% and 25% increase, respectively (*P* ≤ 0.05)). A one‐way ANOVA analysis was performed; error bars represent standard deviation; *n* = 3 biological replicates; *P ≤ 0.05.

In order to confirm that the encapsulated LRP nanoparticles did indeed enter the cells and that the LRP protein was released, HEK‐293 cells were treated for 48 h with 1% and 5% (v/v) empty and LRP nanoparticles and western blot analysis was performed. Densitrometric analysis showed a significant increase for both the 1% and 5% encapsulated LRP treatments (22% and 25% increase, respectively) (Fig. 9). This result shows that the treatment increases overall LRP levels, therefore, confirming that the cells had taken up the nanoparticles, and that the protein was released.

Strangely, a 20% decrease in LRP levels was also detected in the 1% empty nanocapsule treatment (Fig. 9). This could be due to surface interactions with the nanocapsules as the most likely route of internalisation would be phagocytosis or endocytosis [33, 34, 35]. This could cause the internalisation, and subsequent degradation, of the cell surface LRP after the inclusion of the nanocapsules.

**Fig. 10:**
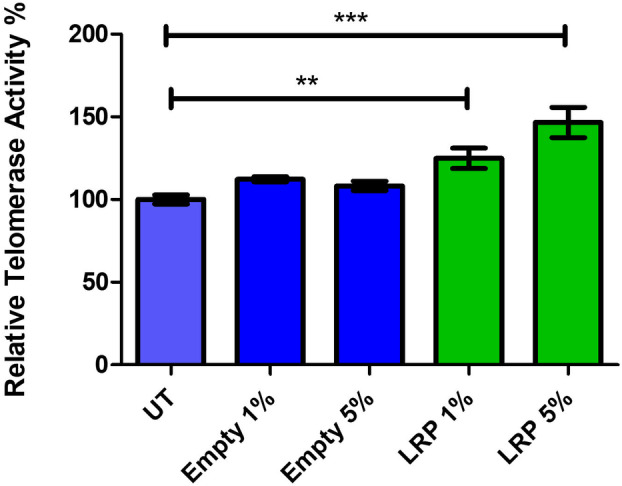
Laminin receptor precursor (LRP)‐nanocapsule treatment elevates telomerase activity *in vitro*. HEK‐293 cells were treated with 1% and 5% (v/v) of both empty and encapsulated‐LRP poly(lactic‐co‐glycolic acid) (PLGA) nanoparticles. The modified telomeric repeat amplification protocol (TRAP) assay was performed by quantitative polymerase chain reaction (qPCR) to determine the effects of the PLGA treatments on telomerase activity. The empty nanoparticle control treatments had no significant effect on telomerase activity, whereas the encapsulated LRP treatments significantly increased telomerase activity for both 1% and 5% treatments by 24.85% (**P ≤ 0.01) and 46.56% (*P* ≤ 0.001), respectively. A one‐way ANOVA analysis was performed; error bars represent standard deviation; *n* = 3 biological replicates; **P ≤ 0.01, ***P ≤ 0.001.

Since we have established a link between LRP and telomerase activity, where an increase in LRP::FLAG expression resulted in a concomitant increase in telomerase activity [6, 10, 11], we therefore subsequently evaluated whether the encapsulated LRP treatments would increase telomerase activity in the HEK‐293 cells. This was done using the modified TRAP assay by qPCR and revealed that telomerase activity significantly increased for both the 1% and 5% encapsulated LRP treatments, with a respective increase of 24.85% (*P* = 0.002) and 46.56% (*P* < 0.0001), while both the 1% and 5% empty nanocapsule treatments did not significantly affect telomerase activity (Fig. 10).

This corroborates our previous findings in which LRP overexpression studies also found an increase in telomerase activity [6, 10, 11]. This increase in telomerase activity alongside the elevated cell viability post‐LRP encapsulated treatment may consequently lead to a comparable rescuing effect from cellular senescence and cytotoxic agents such as Aβ as previously observed. This will therefore allow for further research into using LRP as a therapeutic *in vivo* for the treatment of ageing and age‐related disorders.

Our results therefore suggest that PLGA is a suitable delivery method for protein‐based therapeutics. Identifying an appropriate delivery method for a potential therapeutic molecule is crucial, as unprotected molecules may be degraded by the body *in vivo*, leading to reduced efficacy or nonspecific side effects.

Key advantages of using the PLGA nanocapsules include (a) protection against chemical and enzymatic degradation, thereby increasing stability by increasing the half‐life of the encapsulated drug [36]; (b) optimising the nanocapsule size allows for bypassing cell membranes and the blood–brain barrier; (c) the PLGA polymer is biodegradable into lactic and glycolic acid constituent monomers [14, 36], allowing for biocompatibility and sustained release; and (d) PLGA nanoparticles can be surface modified in various ways to confer organ, tissue or cell specificity. This can be done through PEGylation, where polyethylene glycol (PEG) is added to the surface of the nanocapsule [14]. PEG can bind a variety of targeting molecules such as proteins and antibodies, which are very beneficial for the treatment of specific disorders, as there would be an increase in drug availability for targeted tissue sites, thus reducing required administered doses and, at the same time, reducing off‐target effects due to the uptake in nontargeted tissues. Indeed, the PLGA polymer has previously been used in medical procedures as biodegradable (dissolvable) sutures [15] and has been approved by the FDA to be used as carriers for controlled drug delivery, as well as for tissue scaffolds, due to their excellent safety profile and biocompatibility [37], making PLGA a safe and tested compound in medical applications.

## Conclusion

Previous research has shown that telomerase activity is directly affected by the LRP protein, which has been shown to have therapeutic effects in ageing and age‐related disorders. This is theorised to be at least partially due to a protective role conveyed by telomerase after being elevated by increased LRP levels. Previously, this increase in LRP levels has been achieved by plasmid transfection, which is not an ideal treatment option due to potential unintended effects *in vivo*. Therefore, an LRP protein‐based therapeutic option was developed in the present study. However, to deliver a protein‐based drug, a delivery system is required to protect the therapeutic agent from degradation and facilitate the safe uptake into cells. We therefore designed and optimised a PLGA nanocapsule‐based drug delivery system as an effective method for delivering LRP protein. It was shown that the PLGA nanocapsules alone had no significant effect, while the PLGA encapsulated LRP drug treatment significantly increased cell viability, LRP levels and telomerase activity as previously observed using the LRP::FLAG construct. Ultimately, this has allowed for the translation and progression of our research to *in vivo* models. Thus, in‐line with the wide range of applications that we have shown for elevating LRP levels, this patented LRP‐based therapeutic delivery system could potentially aid in the treatment of age‐related diseases including neurodegenerative disorders, through its ability to increase telomerase activity.

## Conflict of interest

The authors declare no conflict of interest.

### Peer review

The peer review history for this article is available at https://www.webofscience.com/api/gateway/wos/peer‐review/10.1002/2211‐5463.13809.

## Author contributions

MB, TCO, GM, EF and SFTW conceived and designed the project. MB, CM, SG, TCO and MJB acquired the data. MB, MJB, TCO and CM interpreted the data. MB, MJB, TCO and EF wrote the manuscript.

## Supporting information


**Fig. S1.** SEM micrograph of protein encapsulated PLGA nanoparticles.

## Data Availability

The data that support the findings of this study are available from the corresponding author [eloise.vandermerwe@wits.ac.za] upon reasonable request.
