# Supplementary material for: PLGA nanocapsules as a delivery system for a recombinant LRP‐based therapeutic
Source: FEBS Open Bio. 2024 May 3;14(7):1072–86. doi: 10.1002/2211-5463.13809 (PMC11216925; doi:10.1002/2211-5463.13809)
Supplement: Supplementary file 1 — Fig. S1. SEM micrograph of protein encapsulated PLGA nanoparticles. [file FEB4-14-1072-s001.docx]

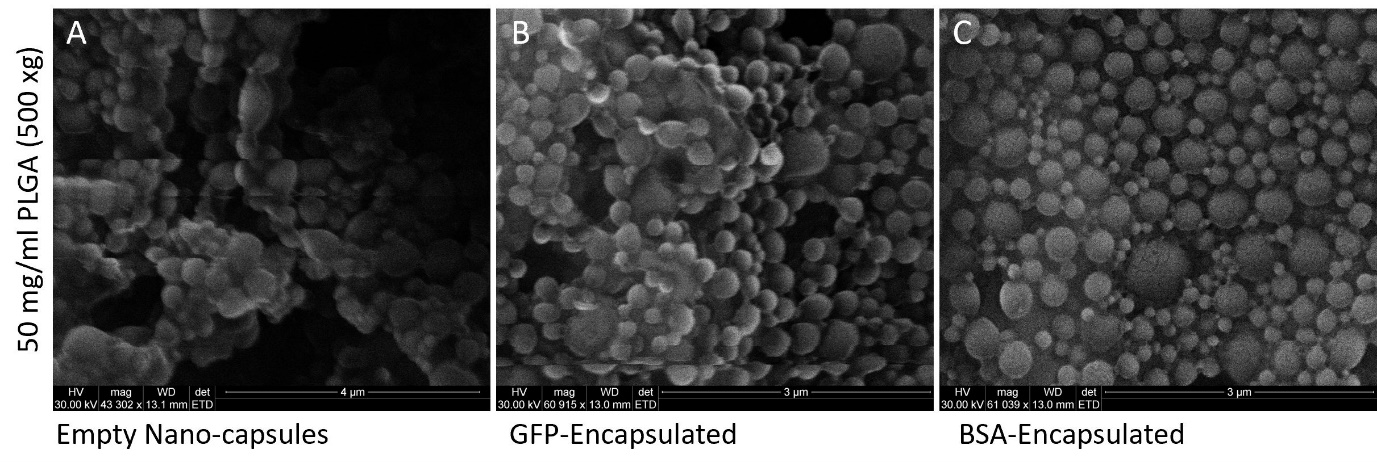


**Fig. SI 1: SEM micrograph of protein encapsulated PLGA nanoparticles**

Scanning electron microscope (SEM) micrographs depicting 500 xg isolated empty and protein encapsulated poly(lactic-co-glycolic acid (PLGA) nanocapsules, synthesised using ethyl acetate (EA) and vitamin-E (TPGS) at 50 mg/ml initial PLGA concentration, (carbon coated - FEI Quanta FEG-SEM - Wits Microscopy and Microanalysis Unit). The morphology of an empty nanoparticle control (A) was compared to successfully encapsulated GFP (B) and BSA (C). (n=3, Scale bars represent 4 µm, 2 µm and 3 µm respectively)
